# Dynamic *in situ* observation of voltage-driven repeatable magnetization reversal at room temperature

**DOI:** 10.1038/srep23696

**Published:** 2016-03-31

**Authors:** Ya Gao, Jia-Mian Hu, C. T. Nelson, T. N. Yang, Y. Shen, L. Q. Chen, R. Ramesh, C. W. Nan

**Affiliations:** 1 School of Materials Science and Engineering, and State Key Lab of New Ceramics and Fine Processing, Tsinghua University, Beijing 100084, China; 2 Department of Materials Science and Engineering, Pennsylvania State University, University Park, Pennsylvania 16802, USA; 3 Department of Materials Science and Engineering, University of California, Berkeley, California 94720, USA

## Abstract

Purely voltage-driven, repeatable magnetization reversal provides a tantalizing potential for the development of spintronic devices with a minimum amount of power consumption. Substantial progress has been made in this subject especially on magnetic/ferroelectric heterostructures. Here, we report the *in situ* observation of such phenomenon in a NiFe thin film grown directly on a rhombohedral Pb(Mg_1/3_Nb_2/3_)_0.7_Ti_0.3_O_3_(PMN-PT) ferroelectric crystal. Under a cyclic voltage applied perpendicular to the PMN-PT without a magnetic field, the local magnetization of NiFe can be repetitively reversed through an out-of-plane excursion and then back into the plane. Using phase field simulations we interpret magnetization reversal as a synergistic effect of the metastable ferroelastic switching in the PMN-PT and an electrically rotatable local exchange bias field arising from the heterogeneously distributed NiO clusters at the interface.

In state-of-the-art spintronic devices, magnetization reversal is driven by current-induced magnetic fields or spin torques^1^. However, the Joule heating from currents can be detrimental to device miniaturization. Directly using a voltage (electric field) to reverse the magnetization is a fundamental solution because the current is ideally zero^2^. Of technological interest, magnetization switching can be achieved in layered magnetic/ferroelectric heterostructures^3^,^4^ with low electric fields of the order of ferroelectric coercivity via electrically driven changes in polarization charges^5^,^6^,^7^,^8^, orbital configuration^9^,^10^,^11^,^12^,^13^, strain^14^,^15^,^16^,^17^,^18^, and/or exchange interaction^19^,^20^,^21^,^22^,^23^,^24^, across the interface. Particularly, a purely voltage-driven net magnetization reversal has recently been observed by photoemission electron microscopy in Co_0.9_Fe_0.1_/BiFeO_3_ heterostructure^24^, based on the electrical reversal of interface weak magnetic moment that is mutually orthogonal to both polarization vector and antiferromagnetic axis in the multiferroic BiFeO_3_ film.

In conventional magnetic/ferroelectric heterostructures, however, purely voltage-driven repeatable magnetization reversal has not yet been experimentally observed in spite of several interesting proposals^25^,^26^,^27^. Relevant experimental efforts involve the use of a magnetic field to either assist the reversal or reset the magnetization to the initial state. For example, Ghidini *et al.*^28^ observed, via magnetic force microscope (MFM), an electrically driven repeatable magnetization reversal via precession magnetization switching in Ni electrodes within commercial multilayer BaTiO_3_-ceramic capacitors, with the presence of a stray magnetic field that is perpendicularly to the precession plane. Liu *et al.*^29^ reported, based on vibrating sample magnetometer (VSM) measurement, an irreversible voltage-induced more than 90° magnetization switching in an antiferromagnetic/ferromagnetic multilayer of FeMn/Ni_80_Fe_20_/FeGaB grown on a (011)-oriented PZN-PT (lead zinc niobate-lead titanate), wherein the antiferromagnetic layer provides a static bias magnetic field to reverse magnetization and a dynamic magnetic field is required to reset the magnetization. Recently, Yang *et al.*^30^ observed, via anisotropic magnetoresistance (AMR) measurement, an irreversible magnetization reversal in an amorphous Co film when applying an in-plane electric field across the ferroelectric Pb(Mg_1/3_Nb_2/3_)_0.7_Ti_0.3_O_3_ (PMN-PT), and the reversal cannot be repeatable before applying an external static magnetic field. More recently, Lee *et al.*^31^ and Bibes *et al.*^32^ reported electrical modulation of magnetism in the FeRh/Ferroelectric single crystal heterostructures, in which the antiferromagnetic and ferromagnetic phase transition of the FeRh can be manipulated by electric field induced piezostrain in the PMN-PT^31^ or BaTiO_3_ (ref. 32) substrate underneath, indicating electric control of magnetization states in these heterostructures.

Here, we report an *in situ* observation of a dynamic, purely voltage-driven repeatable local magnetization reversal (magnetization direction switching) in a simple magnetic/ferroelectric heterostructure consisting of a polycrystalline NiFe film (10 nm) sputtered on a (110)-oriented rhombohedral PMN-PT substrate. Figure 1a shows the schematic of the heterostructure and the measurement geometry. Magneto-optical Kerr microscopy is utilized to acquire the local in-plane magnetization distribution (see Method Section). Unlike previous VSM or AMR measurements^29^,^30^, no driving magnetic field is applied during the characterization process. This purely voltage-driven magnetization reversal is achieved based on an off-plane excursion of magnetization vector induced by the metastable ferroelastic switching in rhombohedral ferroelectrics, plus a local exchange bias field arising from the antiferromagnetic-type clusters at the interface that eventually reverses the in-plane magnetization component.

## Results

**Figure 1:**
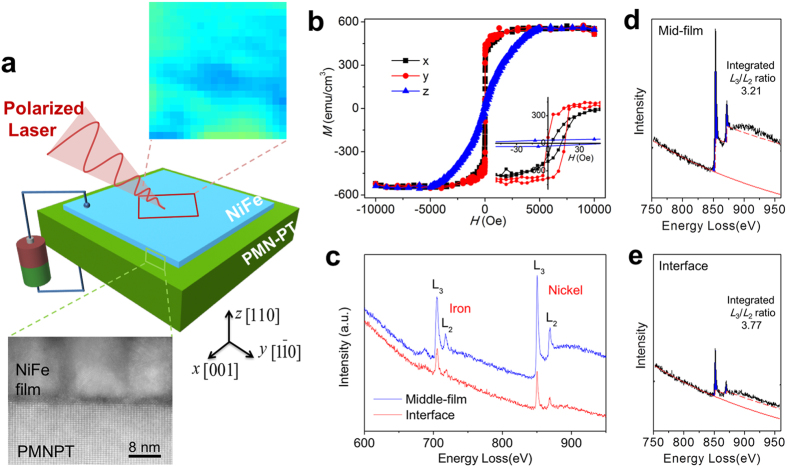
Multiferroic magnetic-ferroelectric heterostructure. (**a)** Schematics of a simple NiFe/(110)PMN-PT bilayer. Upon applying a DC voltage across the bilayer, the in-plane magnetic domain structures (e.g., the top enlarged image) in the NiFe film are mapped out *in situ* by scanning a focused polarized laser (pink cone) from the magneto-optical Kerr microscope. The bottom enlarged area is a HAADF–*Z* contrast image of the heterostructure across the interface. (**b**) In-plane and out-of-plane magnetic hysteresis loops of the bilayer. (**c**) *L*-edge EELS spectra over an extended energy range for Fe and Ni, showing strong *L*_23 _white lines. (**d**,**e**) The *L*_23_ edge spectra from the middle-film and the interface, see Fig. S1-2.

The (110) PMN-PT, which exhibits eight spontaneous polarizations directions along the pseudocubic <111> axes, was pre-polarized against the bottom electrode (i.e., along the positive *z* axis) with a saturation voltage of ~200 V before the magnetic thin film growth. The magnetic hysteresis loops (Fig. 1b) of the heterostructure clearly demonstrate a favorable in-plane magnetic domain alignment in the NiFe film, and weak in-plane exchange bias, i.e., (*H*_EB-x_, *H*_EB-y_) ≈ (7.7 Oe, 8.2 Oe), where *H*_EB-x_ and *H*_EB-y_ are the average bias fields along the *x* and *y* axes, respectively. The bias fields result from a heterogeneously distributed antiferromagnetic-type NiO at the NiFe/PMN-PT interface. The presence of NiO is confirmed by high-angle annular dark field (HAADF) imaging (Fig. 1a), element and valence analysis via energy dispersive X-ray spectrometry (EDS) and electron energy loss spectroscopy (EELS). The EDS results indicate the presence of oxygen-rich top surface and interface in the heterostructure (see Supplementary Information S1). The valence of the metallic elements is studied through EELS^33^ (Fig. 1c) collected inside the NiFe film and at the interface. As we known, the *L*_*3*_ and *L*_*2*_ lines correspond to the transitions from 2p^3/2^ to 3d^3/2^/3d^5/2^ and from 2p^1/2^ to 3d^3/2^, respectively. The *L*_*3*_*/L*_*2*_ ratio values increase with enhancement of the valence state of Ni, which are close to 3.1 for charge-neutral Ni^0^ (ref. 34) and 4.0 for Ni^2+^ in NiO, respectively. Therefore, the increasing of *L*_*3*_*/L*_*2*_ ratio from 3.21 to 3.77 in our study (Fig. 1d) indicated that the valence of Ni at the interface is higher than zero. This is in agreement with the elemental mapping of oxygen as shown in Fig. S1-1. Consequently, we can confirm that NiO exists at the interface. In addition, the ratio of *L*_3_/*L*_2_ collected in another region of the sample demonstrates a change from 3.42 to 3.92 (see Fig. S1-2), providing further evidence for a heterogeneous distribution of NiO at the interface and may exist in the form of clusters, which is indicated by the deviation of *L*_*3*_*/L*_*2*_ ratio from 4.0.

During the poling procedure, a voltage bias is applied perpendicular to the (110) PMN-PT surface, which tends to switch the polarization vector back and forth between the upward 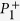
(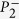) and downward 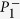
(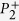) directions (see schematics in Supplementary Information S2), that is, either 180° ferroelectric or 109° ferroelastic switching under a voltage higher than coercive voltage. In addition, an incomplete polarization switching between an out-of-plane *P*_1_(*P*_2_) vector and an in-plane *P*_3_(*P*_4_) vector is also possible when the applied voltage bias is in the vicinity of coercive voltage.

**Figure 2:**
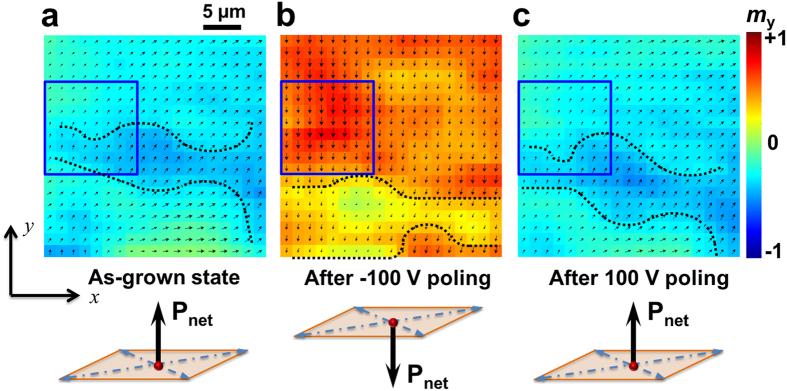
Non-volatile, reversible, and deterministic magnetization reversal driven by polarization reversal. Captured in-plane magnetization distributions (reconstructed Kerr images) within a 30 × 30 μm^2^ range of the NiFe film, (**a**) in the as-grown state, (**b**) after a −100 V poling, and (**c**) a subsequent 100 V poling with their net polarization directions shown below. The dotted lines indicate the positions of the domain walls. All the images are acquired under zero voltage bias with no applied magnetic field. (color bar) *m*_y_ denotes the normalized magnetization along the +*y* direction.

Through a combination of both the longitudinal (*x* axis) and transverse (*y* axis) Kerr microscopic images of the NiFe film after applying and subsequently turning-off voltages (see Method Section), the in-plane magnetic vector diagrams can be reconstructed, as typically illustrated in Fig. 2a–c. In the as-grown film with upward net pre-polarization as shown in Fig. 2a, the magnetic vectors tend to align towards the direction of (+*x*, +*y*) with an average magnetization (*m*_x_, *m*_y_) of about (0.25, 0.28) in a 30 × 30 μm^2^ area, though they are basically non-uniform with well-defined boundaries (domain walls) across the whole region (see the dark dotted line in Fig. 2a). Such an initial magnetization distribution corresponds to the average exchange bias fields of (*H*_EB-x_, *H*_EB-y_) ≈ (7.7 Oe, 8.2 Oe) (Fig. 1c). The unsaturated in-plane magnetization components, represented by both the length of vector arrows and the color bar, are due to a non-zero out-of-plane magnetization component. We attribute this to the presence of in-plane residual strains in the NiFe film (see Supplementary Information S3). Correspondingly, the uniaxial out-of-plane magnetoelastic anisotropy *K*_OOP_ is calculated^35^ to be ~75 kJ/m^3^, which is smaller than the shape anisotropy *K*_shape_ of ~190 kJ/m^3 ^but sufficient to stabilize a fraction of out-of-plane magnetic domains in the polycrystalline NiFe film^36^.

By applying a voltage of −100 V (i.e., toward the bottom electrode) across the PMN-PT to reverse the out-of-plane net polarization component and subsequently turned off, surprisingly, most magnetic vectors rotate by >135° to align along -*y* axis (see Fig. 2b) except some tilting toward -*x* axis near the boundary (the dark dotted lines), with an average in-plane magnetization (*m*_x_, *m*_y_) of about (−0.052, −0.47), (the magnetization values are normalized to (−1, 1), details in method section). Moreover, the boundaries move remarkably towards the -*y* direction probably due to the movement of its elastically coupled ferroelectric domain (wall) underneath^37^. Very interestingly, the NiFe film can return to be almost exactly the same as the initial magnetic domain state [average (*m*_x_, *m*_y_) = (0.25, 0.25), see Fig. 2c] after a subsequent +100 V poling albeit with a small shift and curving for the boundaries. Thus, we have directly observed a non-volatile and repeatable local magnetization reversal occurs purely driven by polarization reversal (the repeated results are shown in Fig. S4-1). Similar reversal behaviors have also been observed in other regions of the NiFe film (see Supplementary Information Fig. S4-2).

**Figure 3:**
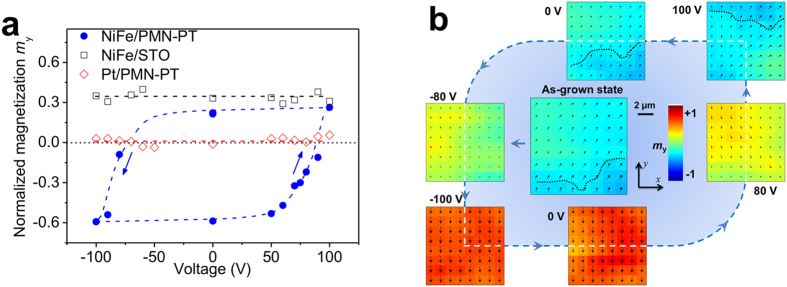
Purely voltage-driven bistable magnetization reversal. **(a)** Normalized magnetization *m*_y_ of the NiFe film as a function of voltage for one local region of 12 × 12 μm^2^ (i.e., the blue square region in Fig. 2) in the NiFe/PMN-PT, as well as the NiFe/STO and the Pt/PMN-PT heterostructures for comparison. A positive (negative) *m*_y_ corresponds to an upward (downward) average polarization (the insets) in the bottom PMN-PT layer. (**b**) Typical in-plane magnetization distributions captured during the voltage sweeping. The dotted line indicates the position of domain wall.

For clearer illustration, we focus on a local region of 12 × 12 μm^2^ around the boundary, i.e., the square region with solid frame shown in Fig. 2a–c. The change in the normalized magnetization *m*_y_ of this region with voltage (*V*) presents a hysteresis loop (Fig. 3a). Corresponding to an upward or downward net polarization in the bottom (110) PMN-PT layer, the upper NiFe film shows either a positive (+*y* axis) or negative (−*y*) normalized magnetization *m*_y_ at zero voltage bias, clearly indicating a non-volatile voltage-driven magnetization reversal. In particular, at around the coercive voltage (about ±80 V, see ferroelectric hysteresis loop of PMN-PT crystal in Fig. S2a), the magnetization in the investigated area is very likely to be pointing out-of-plane, because both *m*_*x*_ and *m*_*y*_ are almost zero and the local magnetization vectors in that area (12 × 12 μm^2^) would be largely uniform (single-domain). Similar *m*_y_ − *V* hysteresis loop has also been observed for the second applied voltage cycle (see Supplementary Fig. S5). These magnetic vector diagrams of the local region of 12 × 12 μm^2^ acquired by sweeping the voltages (Fig. 3b and also Fig. S5) also provide direct evidence of such magnetization reversal. When the voltage increases to −80 V (i.e., around the coercive voltage), the upward pre-polarization vectors 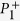/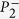 in the PMN-PT would be switched to the film plane, i.e., 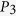/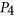, producing ferroelastic tensile strain along the *y* axis and shear strain within the *xy* plane (as discussed in Supplementary Information S2). Such ferroelastic strains are superimposed on the residual strain, and together induce a dominant out-of-plane magnetization moment in the local region (see the image at −80 V in Fig. 3b). When the voltage further increases to −100 V, the net polarization starts to grow downward when the polarization vector rotates from the in-plane 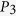/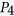 to the out-of-plane downward 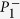/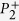 direction (Fig. S2b), producing ferroelastic strains of opposite signs. As a result, the magnetization vector would be switched back towards the film plane, and remarkably, it would reorient along the -*y* direction. Such an almost uniform magnetization remains stable after turning off the voltage (i.e., 0 V), though it would somewhat tilt towards out-of-plane with slightly smaller *m*_y_ (see Fig. 3a and corresponding distribution in Fig. 3b). This is due to ferroelastic relaxation which tends to form 180° 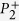/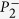 or 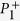/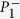 domain walls out of the existing downward 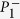/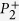 polarizations^38^. Furthermore, by applying a positive voltage bias, the (0, –*y*) magnetization distribution can be switched back to the initial (+*x*, +*y*) magnetization state by over 135°, following quite similar paths, i.e., first being out-of-plane in the vicinity of the coercive field (80 V), and then back to the film plane with an reorientation under 100 V and the subsequent 0 V.

For comparison, we also did the same measurements on similar heterostructures of Pt(10 nm)/PMN-PT and NiFe(10 nm)/SrTiO_3_, as plotted in Fig. 3a. For the non-magnetic Pt on PMN-PT, there are no appreciable magnetic signals being detected; while for the same NiFe film on SrTiO_3_, there is no reversal of the *m*_y_ during a bipolar voltage cycle in spite of slight fluctuations. The corresponding scanning Kerr images for these two heterostructures are shown in Supplementary Information S6. This comparison further justifies that our observations of non-volatile, purely voltage-driven local magnetization reversal in the NiFe film grown on PMN-PT do not arise from any other side effects.

Since rotating the upward polarization vectors 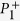
(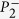) to the downward 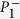
(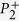) (and vice versa, see Fig. S2b) would not impose any additional structural transformation within the (110) plane of the PMN-PT, the observed bistable magnetization states, i.e., the (+*x*, +*y*) state under the upward net polarization and the (0, −*y*) state under the downward net polarization, should have almost identical strain states. Therefore, such bistable magnetization reversal in the magnetic thin film is not directly determined by the bistable electric-field-induced strains^39^,^40^ but likely by a combination of the following two features. First, the electric-field-induced strain controls the position of magnetic domain walls that are elastically coupled to the ferroelastic walls underneath^41^. For example, the in-plane magnetic domain walls are moved away from the local 12 × 12 μm^2^ region with the upward 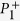
(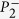) to the in-plane *P*_3_(*P*_4_) ferroelastic switching (e.g. 0 V to −80 V), while reestablish for a backward *P*_3_(*P*_4_) to 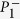
(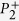) switching (e.g., 80 V to 100 V). Second, an exchange-bias effect could be expected due to the antiferromagnetic NiO clusters existing at the interface. Therefore, through a combination of the elastically coupled domain walls of NiFe and PMN-PT, with the ferromagnetic-antiferromagnetic exchange coupling between the NiFe and interface NiO clusters, a local exchange bias field *H*_EB_ could be expected and subsequently induces a magnetization reversal. Based on the measured magnetic vector diagrams (Fig. 3b and also Fig. S5), it is reasonable to assume that, for the present local region of 12 × 12 μm^2^, the local *H*_EB_ is along the (+*x*, +*y*) direction when the domain walls (see the dotted line in the images of 0 V and 100 V) present in the local region, while rotates to be along the (0, −*y*) directions when the domain walls move away (Fig. 3b). Nevertheless, given the heterogeneous nature of the local *H*_EB_, local voltage-driven magnetization switching paths are not limited to the 135° reversal illustrated above. Indeed, we have also observed voltage-driven 45°, 90°, even almost 180° magnetization switching in other local regions of this sample (See Supplementary Information S7).

**Figure 4:**
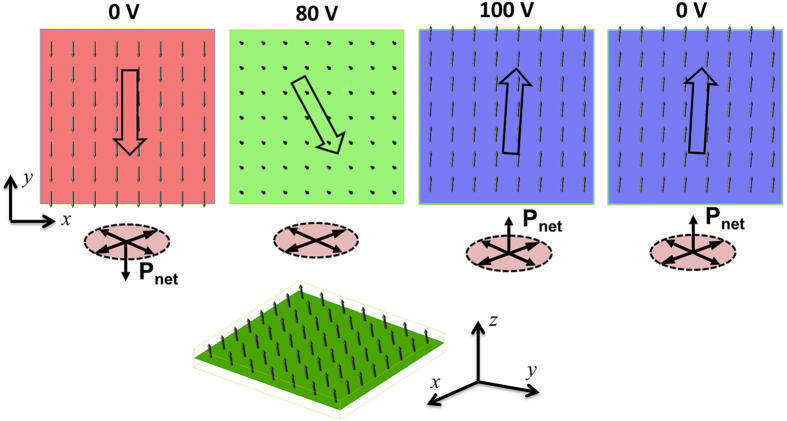
Interpretation of the observed magnetization switching path with phase-field simulations. Local magnetization distributions of the NiFe, upon applying perpendicular voltages across the PMN-PT to reverse the net polarization from a downward direction (0 V on the left) first to the plane (80 V), then to the upward direction (100 V) and its subsequent remnant state (0 V on the right). The three-dimensional image of the magnetization vectors for 80 V is shown at the bottom.

To further understand this plausible explanation of strain and exchange-bias co-mediated magnetization reversal, phase-field modeling (see Methods) was employed to simulate the evolution of local magnetization distributions when increasing the voltage from 0 V with a downward net polarization, to 80 V and 100 V, and then back to 0 V with an upward net polarization. As shown in Fig. 4, the simulated results mostly reproduce the observed magnetization reversal path, e.g., see the simulated three-dimensional tilting magnetization distribution at 80 V. A magnetization reversal of ~167° is obtained compared to the experimentally observed 135° reversal. Such difference could arise from the local inhomogeneity in the experimentally measured distributions. Interestingly, our simulations also indicate that the experimentally observed local magnetization reversal in the present region (Fig. 3b) may correspond to a specific polarization switching path from 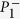
(0 V) to *P*_3_ (80 V), then to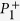 (100 V), followed by the nucleation of 180° domain wall of 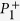
**/**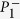 at remnant state (0 V). Detailed discussions are also shown in Supplementary Information S8.

## Discussion

This work proposes a promising, new pathway towards purely voltage-driven repeatable magnetization reversal based on a simple magnetic/ferroelectric bilayer. Upon applying a dynamic voltage perpendicularly to the ferroelectric layer, we have observed *in situ* that the magnetization at first experiences an off-plane excursion, then goes back to the film plane with a reversal of in-plane magnetization component. Such a unique magnetization switching path is due to the synergistic effect of two important kinetic processes across the heterostructure interface: (1) the out-of-plane to in-plane domain switching in rhombohedral ferroelectrics generates a local strain to switch the local magnetic domains from in-plane to out-of-plane orientation; (2) the local exchange bias field at the interface, arising between the heterogeneously distributed antiferromagnetic NiO clusters and the ferromagnetic NiFe film, also changes associated with the out-of-plane switching of local magnetic domains. When local strain relaxes upon the completion of ferroelectric domain reversal such that the out-of-plane magnetic domains are ready to fall back to the film plane due to demagnetization, the aforementioned unidirectional local exchange bias field can stabilize the local magnetic domain to the opposing in-plane direction rather than its initial in-plane direction. The reversal of local magnetic domains is therefore completed. This unique switching path is demonstrated using phase-field simulations (Fig. 4). The presence of the NiO clusters is confirmed by EELS and EDS measurements, yet, it remains unclear how and why these NiO clusters form around the interface and how the nature of interface changes in response to applied electric field. These are all important issues remaining to be explored in the future. It is worth mentioning that the above-mentioned electrically tunable exchange bias field have been reported in various other interfaces with coexistence of magnetic, antiferromagnetic, and ferroelectric (electric) orders [e.g., CoFe/BFO^23^, NiFe/LuMnO_3_^42^, (La,Sr)MnO_3_/BFO^43^, CoPd/Cr_2_O_3_^44^]. The stability of the potential device based on the present heterostructure largely relies on how many cycles that the ferroelectric domain switching can last without appreciable failure. Specifically, for PMN-PT-based, repeatable voltage-controlled magnetic properties has been demonstrated for up to thousands of cycles without evident degradation in heterostructures with magnetic films on PMN-PT single crystals^40^,^45^. Even higher stability can be expected if only piezostrains are utilized to control magnetism without requiring ferroelectric domain switching. However, in order to fully understand the physics involved in the present observations, further studies are needed to unravel the complex interplay between the antiferromagnetic, ferroelectric, elastic, structural (e.g., grain boundaries) orders, and maybe the polarization charges at the present NiFe/(110) PMN-PT interface^46^. We thus hope our observation could stimulate more research efforts both fundamentally and experimentally.

## Method Section

### Samples

The 10 nm NiFe thin film was grown on a 2 mm × 2 mm × 0.5 mm (110) PMN-PT single-crystal layer by magnetron sputtering. The base vacuum was 8 × 10^−7^ torr, and the growth was carried out at 200 °C for the substrate under 0.002 torr of argon pressure, with an input DC power of 60 W for the target.

### Structural characterization and element analysis

The NiFe/PMN-PT interface was analyzed by transmission electron microscopy (TEM, FEI.). The HAADF-STEM mode was used with the excitation voltage of 80 kV to prevent the electric beam from damaging the films. The *L*-edge spectrum was collected in the highlighted quadrate regions that covered the mid-film and the interface of the heterostructure in the HAADF images during the 120 s exposure time for each measurement. Element and valence analysis were performed via energy dispersive X-ray spectrometry (EDS) and electron energy loss spectroscopy (EELS) in an aberration (Cs) corrected transmission electron microscopy (TEM, FEI., TitanX 60-300 for EDS and TEAM 0.5 for EELS).

### Macroscopic measurements

The magnetic hysteresis loop of the NiFe film was measured by Physical Property Measurement system (PPMS, Quantum Design Inc.), and the piezoelectric response (ferroelectric hysteresis loop) of the PMN-PT single crystal was characterized by Ferroelectric (WS2000, Radiant Technologies, Inc.).

### Dynamic magneto-optical Kerr measurements

The in-plane magnetization distributions in the films were reconstructed by combining the *in situ* acquired longitudinal and transverse Kerr images from the magneto-optical Kerr microscopy. Both longitudinal and transverse Kerr signals were obtained during one scan with each *in situ* applied voltage. The magnetization images were measured in a 30 × 30 μm^2^ scanning region with the pixel of the 1.5 × 1.5 μm^2^. All collected data were normalized within −1 to 1 range by subtracting the center value of each scan and then dividing the range value as the saturated magnetization. The normalized data are corresponding to the magnetization components along the *x* and *y* axes, respectively (see Supplementary Information S9 for an illustration of the data processing). The *in situ* perpendicular voltage was applied by a Source Meter (Keithley Instrument Inc., 2410). The Kerr microscopy image at each voltage was obtained twice, with one measured at one minute after the polarization current (less than 0.001 μA) was stable and the second after turning the applied voltage off. No external magnetic field was applied throughout the characterization process.

### Phase-field simulations

The present phase-field model includes discretized three-dimensional cells of 90Δ*x* × 90Δ*y* × 24Δ*z*. Specifically, the bottom cells of 90Δ*x* × 90Δ*y* × 10Δ*z* and their overlaying cells of 90Δ*x* × 90Δ*y* × 10Δ*z* are designated as the phases of ferroelectric substrate and the magnetic thin film, respectively, and the rest represent the air. By introducing these different phases in the model, the elastic boundary condition of a thin film, i.e., clamped in the film plane and stress-free out of the plane, can be taken into account (see details in ref. 47). A cell size of Δ*x* × Δ*y* × Δ*z* = 11.11 nm × 11.11 nm × 1 nm is utilized to describe a model magnetic thin film with a size of 1 μm × 1 μm × 10 nm. As shown in Fig. 3, the experimentally measured magnetization distributions are largely uniform in orientation within a local region of 12 μm × 12 μm. Therefore, the use of a smaller in-plane size of 1 μm × 1 μm is proper (and saves computation time) for demonstrating the mechanism of magnetization reversal in the entire region of 12 μm × 12 μm. In-plane periodic elastic and magnetostatic boundary conditions are required in this case.

Kinetic evolution of the local magnetization vectors ***M***(***r***) = *M*_s_[*m*_x_(***r***), *m*_y_(***r***), *m*_z_(***r***)] (where *M*_s_ is the saturation magnetization, and ***m*** the normalized magnetization component) and their equilibrium distribution under strain and exchange bias fields are obtained by solving Landau-Lifshitz-Gilbert equation, given in dimensionless units as,


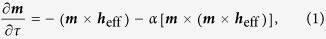


where the time step *τ* relates to the practical time *t* as *τ* = [*γ*_0_M_s_/(1 + *α*^2^)]*t* (ref. 26), where *γ*_0_ and α denote the gyromagnetic ratio and the damping coefficient. These two kinetic coefficients have little influence on equilibrium magnetic domain structures under static external stimuli (e.g., the static strain and static exchange bias field here). The effective magnetic field ***h***_eff_ = −(1/*μ*_0_*M*_s_)(δ*f*_tot_/δ***M***), where *μ*_0_ is the vacuum permeability; *f*_tot_ indicates the total free energy density of the magnetic thin film, which is a sum of the exchange energy density (*f*_ex_), the magnetostatic energy density (*f*_ms_), the elastic energy (*f*_elast_), and the energy density of the exchange bias field [*f*_EB_ = −*μ*_0_*M*_s_ (***H***_EB_ · ***m***)]. Expressions of *f*_ex_, *f*_ms_, and *f*_elast_, along with detailed procedures of solving ***h***_eff_ and the equation (1), are directly given in ref. 47 and references therein. The *M*_s_ of the NiFe film is taken as about 550 kA/m based on PPMS measurement. The other magnetic parameters and elastic parameters of the NiFe film can be found in the supplemental materials of ref. 26.

## Additional Information

**How to cite this article**: Gao, Y. *et al.* Dynamic *in situ* observation of voltage-driven repeatable magnetization reversal at room temperature. *Sci. Rep.* **6**, 23696; doi: 10.1038/srep23696 (2016).

## Supplementary Material

Supplementary Information
