# Supplementary material for: Dynamic in situ observation of voltage-driven repeatable magnetization reversal at room temperature
Source: Sci Rep. 2016 Mar 31;6:23696. doi: 10.1038/srep23696 (PMC4814776; doi:10.1038/srep23696)
Supplement: Supplementary Information [file srep23696-s1.pdf]

Supplementary Information

Dynamic *in situ* observation of purely voltage-driven repeatable magnetization reversal at room temperature

Ya Gao,[1†] Jia-Mian Hu,[2†] C. T. Nelson,[3] T. N. Yang,[2] Y. Shen,[1] L. Q. Chen,[1,2] R. Ramesh,[3]* and C. W. Nan[1]*

[1]*School of Materials Science and Engineering, and State Key Lab of New Ceramics and Fine Processing, Tsinghua University, Beijing 100084, China*

[2]*Department of Materials Science and Engineering, Pennsylvania State University, University Park, Pennsylvania 16802, USA*

[3]*Department of Materials Science and Engineering, University of California, Berkeley, California 94720, USA*

S1. EDS and EELS analysis of the NiFe/PMN-PT heterostructure

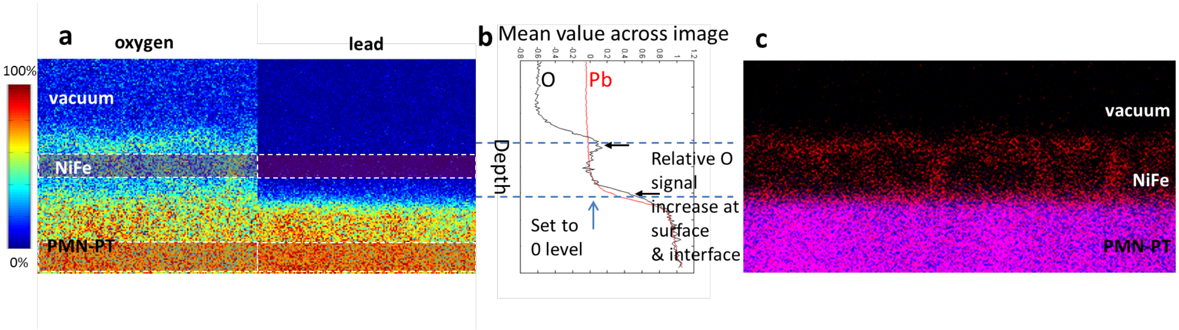

Figure S1-1| (**a**) Normalized and background subtracted X-ray counts for oxygen (left) and lead (right) distribution. (**b**) Mean value of oxygen and lead across the EDS image. (**c**) Normalized color map of oxygen (red) and lead (blue).

The element composition in the heterostructure was measured by EDS. The color maps of net oxygen and lead counts are shown in Fig. S1-1a. As data processing, the shape of the Pb signals is considered as the oxygen background level to remove the contributions from the PMN-PT and the NiFe phases, so that the maps are normalized setting the mean of the highlighted regions (dashed shadow boxes) to 0 and 1, respectively. In fig. S1-1b, the mean value across the EDS images indicates the O relative value increased at the heterostructure's surface and the interface. The normalized maps in Fig. S1-1c show more details of the O distribution, thus, the surface of the sample, the grain boundaries of the polycrystalline NiFe film and the interface of the heterostructure are O rich.

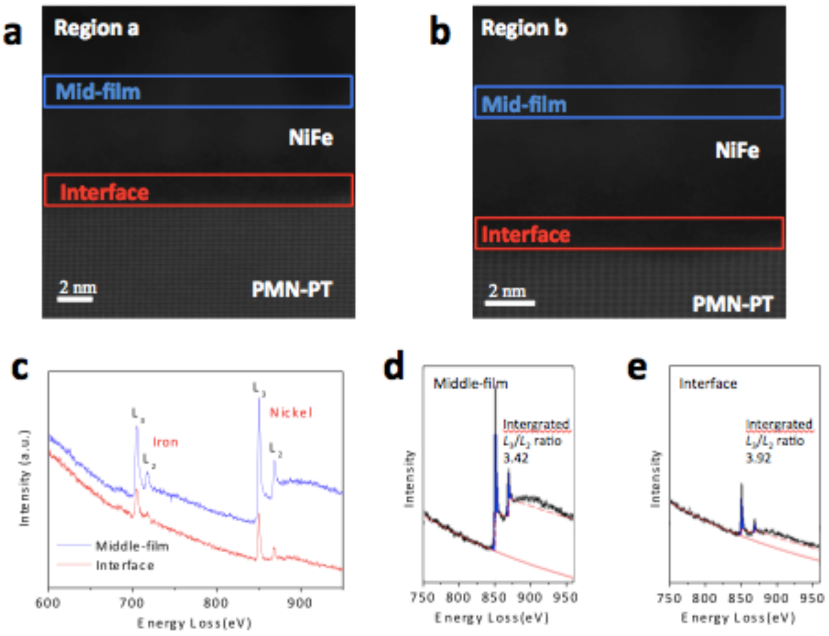

Figure S1-2| The HAADF images of the regions (**a** and **b**) used for EELS spectrum collection. (**c**) L-edge spectra for region b. (**d** and **e**) The L_{23} edge spectra from the mid-film and the interface of region b.

The selected two regions for EELS experiments are shown in Fig. S1-2(a and b). The spectra

are collected in the box regions for mid-film and interface information. The L-edge spectrum for region b is shown in Fig. S1-2c, and detailed white-line ratio L_3/L_2 are 3.42 and 3.92 for mid-film and interface, which is consistent with the results for region a (Fig. 1d-e).

S2. The ferroelectric hysteresis loops and polarization switching paths of the (110) 0.7PMN-0.3PT

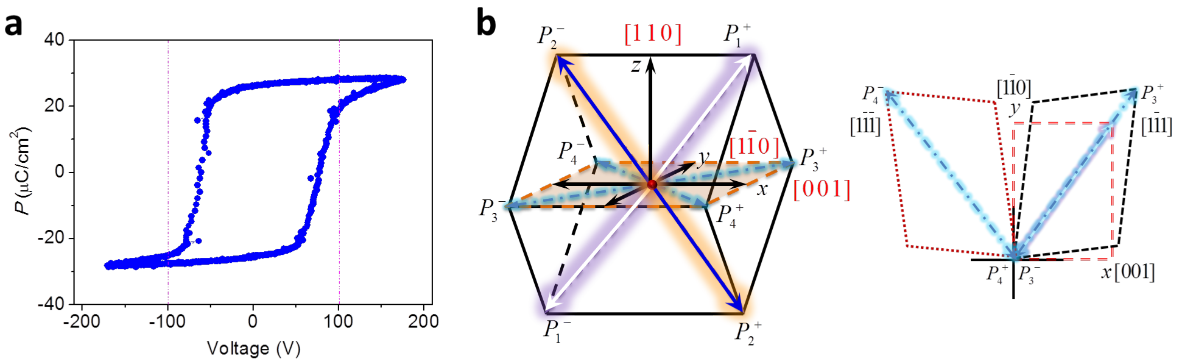

Figure S2 | (**a**) Ferroelectric (FE) hysteresis loop of the (110) rhombohedral 0.7PMN-0.3PT. (**b**) Possible polarization switching paths (upper panel) and the in-plane structural deformation caused by out-of-plane to in-plane switching.

As shown in Figure S2a, a well-defined FE hysteresis loop is observed for the (110) PMN-PT single crystal, with a saturated polarization P_s of about 29.6 μC/cm^2, a remnant polarization P_r of about 27.5 μC/cm^2, and the coercive voltage V_c is around 75.9 V. The discontinuous jumps in the loop are due to the complex domain configurations in such rhombohedral ferroelectrics, which are more remarkable in the vicinity of V_c, indicating the drastic polarization switching process via irreversible ferroelastic domain wall motion. When the applied voltage exceeds ±100 V, the value of polarization starts to become saturated. In this case, the polarization reversal is almost completed where the domain would gradually coarsen via sideway growth

of the domain walls [1].

S3. Calculating the in-plane residual strains

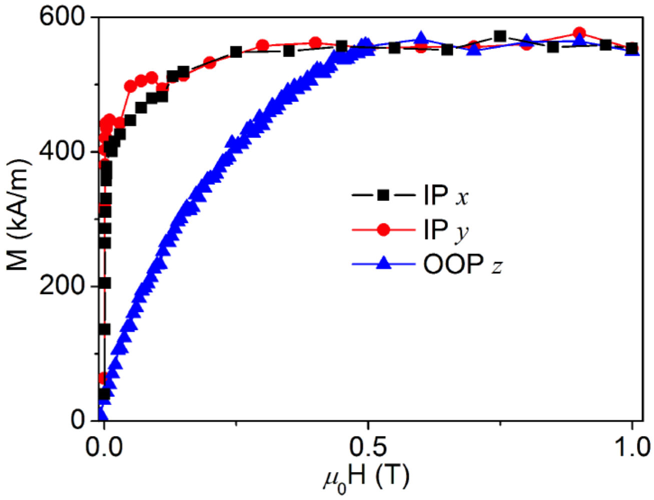

Figure S3| Virgin magnetization curves after demagnetization along the in-plane (IP) x, y, and the out-of-plane (OOP) z axes.

The magnetization curves clearly indicate an out-of-plane hard axis resulting from the large shape anisotropy energy $K_{\text{shape}} = \frac{1}{2}\mu_0 M_s^2$ of about 190 kJ/m^3, where μ_0 and M_s(~550 kA/m) are the vacuum permeability and the saturation magnetization, respectively. For the present polycrystalline NiFe film with negligible magnetocrystalline anisotropy, the magnetoelastic anisotropy $K_{me} = -\frac{3}{2}\lambda_s \sigma_{\text{IP}}$ simply corresponds to the area between the in-plane easy (e.g., x and y) and out-of-plane hard axis (z) virgin magnetization curves [2] as shown in Fig. S3. Here λ_s is the magnetostrictive constant taken as -32.9 ppm as in the polycrystalline Ni-rich NiFe film with negative magnetostriction [3], and σ_{IP} denotes the in-plane residual stress which is normaly assumed to be isotropic (i.e., σ_{xx}=σ_{yy}=σ_{IP}). By comparing the z axis curve to the x and y axes, σ_{IP} can be obtained to be around 1.45 GPa and

1.58 GPa, respectively. Their corresponding in-plane residual strains $\varepsilon_{IP} = \left(s_{11} + s_{12}\right)\sigma_{IP}$ can be further calculated to be 0.668% and 0.73%, respectively, where s_{11} (0.0073 GPa^{-1}) and s_{22} (-0.0027 GPa^{-1}) are the elastic compliance constants. However, the magnetization curves imply a slightly more favorable in-plane *y* easy axis (Fig. S3), which should be attributed to the anisotropic in-plane residual strains ($\varepsilon_{xx} \neq \varepsilon_{yy}$) since no annealing magnetic field is applied during the magnetic thin film growth. Further regarding the negative magnetostriction, ε_{xx} and ε_{yy} should be 0.73% and 0.668%, respectively. Such tensile built-in strains are thus responsible for the observed out-of-plane magnetization component in the film.

S4. The second cycle of the voltage control of SKM images and similar results in another 30×30 μm² region

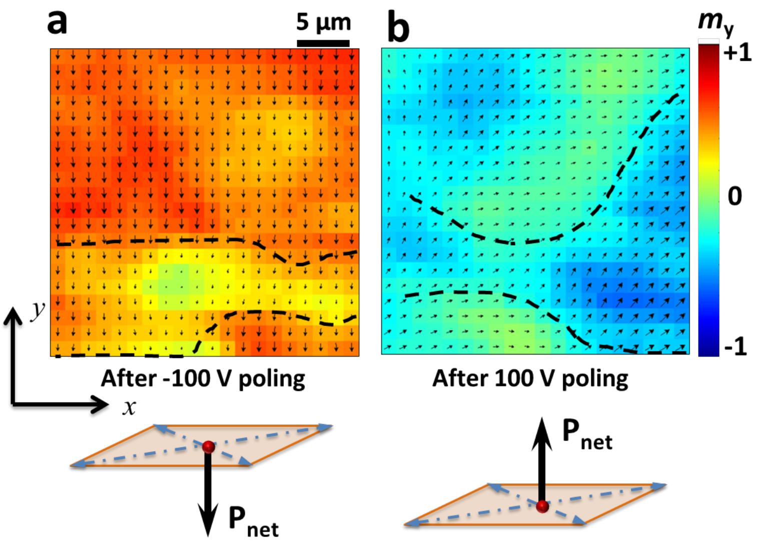

Figure S4-1 | Magnetization reversal driven by polarization reversal during the second poling cycle. **(a)** after a -100 V poling, and **(b)** after a 100 V poling with their net polarization directions shown below.

The voltage driven magnetization reversal was observed in the following cycles of the applied voltage (the second cycle is shown in Fig. S4-1) and other regions of the NiFe film, as shown in Fig. S4-2. Similar shape of the possible magnetic domain walls are also labeled with dashed lines in the figures. In another region of the NiFe film, the magnetization of the as-grown state (Fig. S4-2a) almost point to the (+x, +y) direction. During the first poling with a -100 V, the polarization switched from upwards to downwards, while the magnetization rotated to –y direction (Fig. S4-2b). And the second poling with a +100 V reversed the FE state of the PMN-PT and the magnetization state of the NiFe film (Fig. S4-2c) all back to the as-grown states.

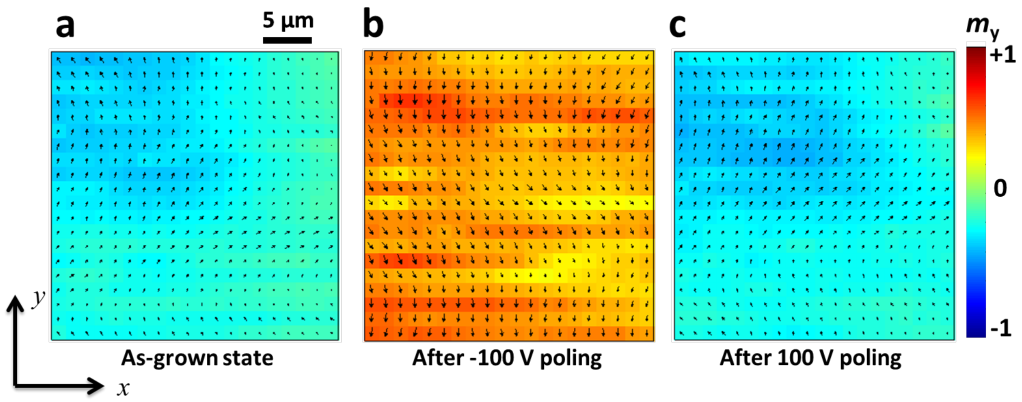

Figure S4-2 | Magnetization reversal driven by polarization reversal in another 30×30 μm^2 region. (**a**) in the as-grown state, (**b**) after a -100 V poling, and (**c**) a subsequent 100 V poling with their net polarization directions shown below.

S5. The m_y– V hysteresis loop for the second applied voltage cycle.

As shown in Fig. S5, the m_y-V loop exhibited hysteresis like shape, and the magnetization vector mapping images during the second applied voltage cycle has similar

switching behavior (see Fig. 3). At the vicinity of the ferroelectric coercive voltage (-80 V), the measured point is away from the curve, which suggests the lag of the ferroelectric domain switching. As shown in Fig. S5, applying the larger-magnitude voltage of -100 V leads to drastic different magnetic state, suggesting a more complete switching of ferroelectric domains.

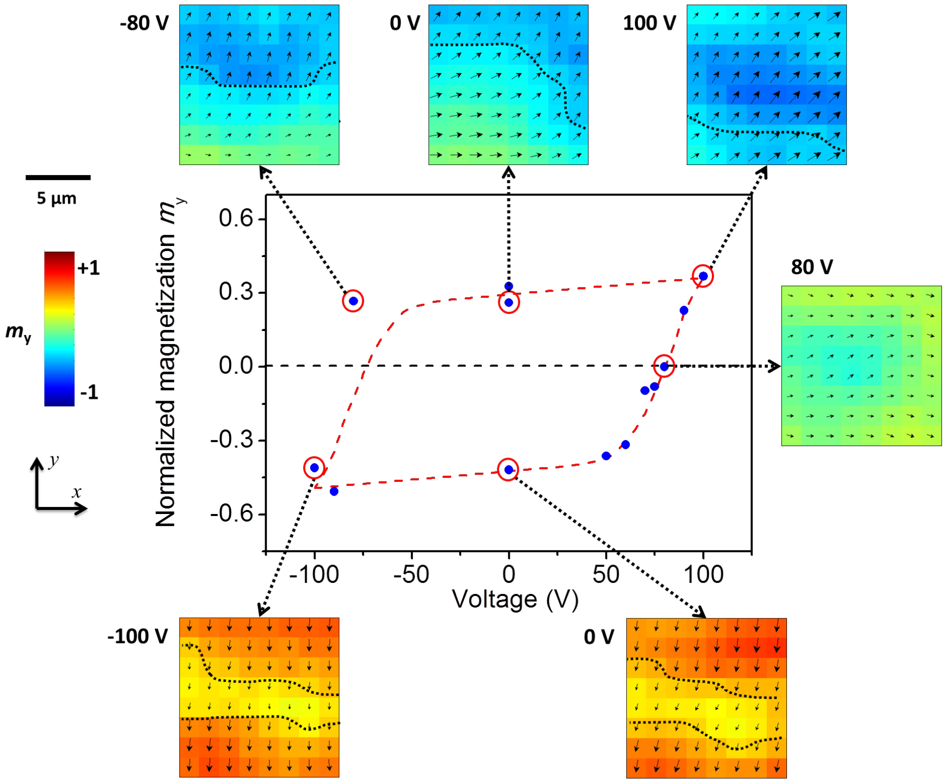

Figure S5 | The m_y–V hysteresis loop and the detailed SKM images for the second applied voltage cycle.

S6. SKM mapping of the Pt/PMN-PT and NiFe/STO heterostructures.

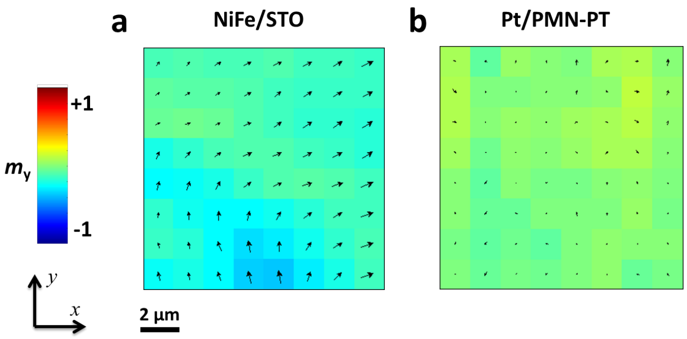

Figure S6 | The as-grown states of the SKM images for (**a**) NiFe/STO and (**b**) Pt/PMN-PT heterostructures.

As a comparative experiment, the FE layer and the FM layer of the coupled heterostructure was changed respectively. The same scanning Kerr signals were collected in 12×12 μm^2 regions for each sample. The image of the NiFe/STO shows magnetization mainly towards the (+x, +y) direction (Fig. S6a), however, no magnetization switching or rotation was observed after an applied voltage on the STO substrate. On the other hand, the mapping of the Pt/PMN-PT sample (Fig. S6b) showed no strong vector in any pixel. The comparisons demonstrate that the magnetization reversal we observed in the NiFe/PMN-PT heterostructure is determined. Moreover, the magnitude of the Pt/PMN-PT images can manifest the noise level of the SKM measurement, which is obviously negligible comparing to the magnetizations.

S7. SKM images of the NiFe/PMN-PT with different magnetization rotation angles.

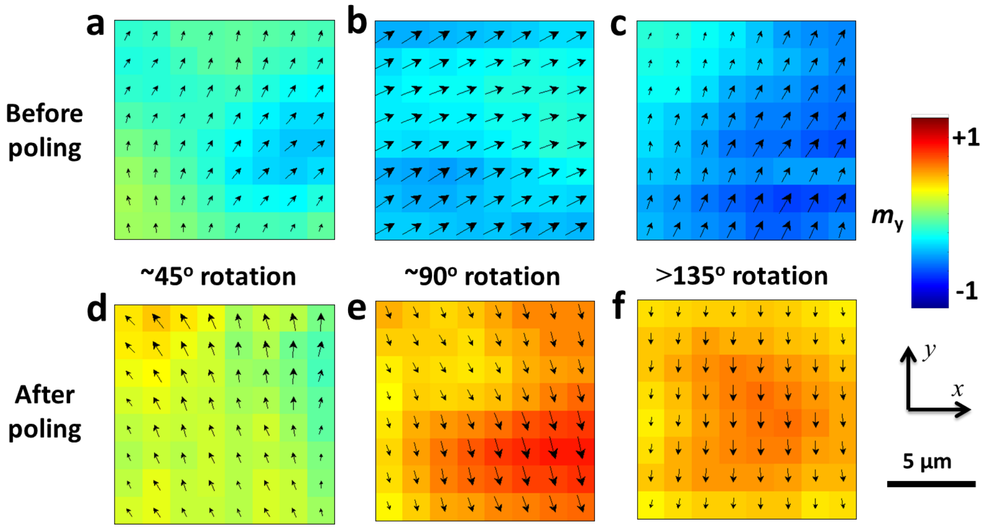

Figure S7 | SKM results in other regions with different rotation angles. The as-grown states (**a** to **c**) and switching states (**d** to **f**) of different areas.

The sample has different regions with different magnetization rotation angles. As shown in Fig. S7, the observed areas exhibited less than 45° rotation (Fig. S7 (a and d), which could result from pinning effect of the adjacent obstacles), 90° rotation (Fig. S7 (b and e)) and even more than 135° rotation (Fig. S7 (c and f)). Such difference is an evidence that the $\boldsymbol{H}_{EB}$ is a heterogeneous field that arises from NiO clusters at the interface of the heterostructure.

S8. Details of the phase-field simulations.

Details of the phase-field models have been reported elsewhere in ref.40 in the main text. The magnetic vector diagrams shown in Figure 4 corresponds to a polarization switching path from P_1^- (0 V) to P_3 (80 V), then to P_1^+ (100 V), followed by possible relaxation of ferroelastic strain (taken as 10% herein) at the remnant state (0 V). Table S1a summarizes the values of ferroelastic strain induced by such a switching path that were superimposed on the built-in residual strain of (ε_{xx}, ε_{yy})=(0.73%,0.67%) during simulations, as well as the

components of local exchange bias fields H_{EB} along the x and y directions. During the calculations of ferroelastic strains, the two remnant states with downward (0 V on the left) and upward (0 V on the right) net polarizations are taken as the reference states with zero ferroelastic strains.

Table S1a

	**0 V**	**80 V**	**100 V**	**0 V**
ε_{xx}	0	0	0	0
ε_{yy}	0	0.1725%	-0.0192%	0
ε_{xy}	0	0.1223%	-0.0136%	0
H_{EB}(x)	0	0	7.7 Oe	7.7 Oe
H_{EB}(y)	-11.25 Oe	-11.25 Oe	8.2 Oe	8.2 Oe

Corresponding values for the polarization switching path from P_1^- to P_4, then to P_1^+ are listed in Table S1b.

Table S1b

	**0 V**	**80 V**	**100 V**	**0 V**
ε_{xx}	0	0	0	0
ε_{yy}	0	0.1725%	-0.0192%	0
ε_{xy}	0	-0.1223%	0.0136%	0
H_{EB}(x)	0	0	7.7 Oe	7.7 Oe
H_{EB}(y)	-11.25 Oe	-11.25 Oe	8.2 Oe	8.2 Oe

Figure S8 further shows the simulated local magnetization distributions with these values. As seen, the changes in the signs of the shear strain ε_{xy} result in different magnetization distributions at 80 V and 100 V. They have opposite in-plane orientations along the x axis compared to the previous case. Such are plausible given that a tensile and a compressive ε_{xy} typically favor a magnetization distribution along the (-x,-y) and (+x, +y) axes, respectively, in a magnetic thin film with negative magnetostriction (e.g., the NiFe herein) according to our previous simulations [4]. Based on the arguments above, we suspect the polarization switching path in the local region underneath the images shown in Figure 2 and 3 is from P_1^- (0 V) to P_3 (80 V), then to P_1^+ (100 V), or equivalently, from P_2^+ (0 V) to P_4 (80 V), then to P_2^- (100 V), as illustrated in Figure 1b.

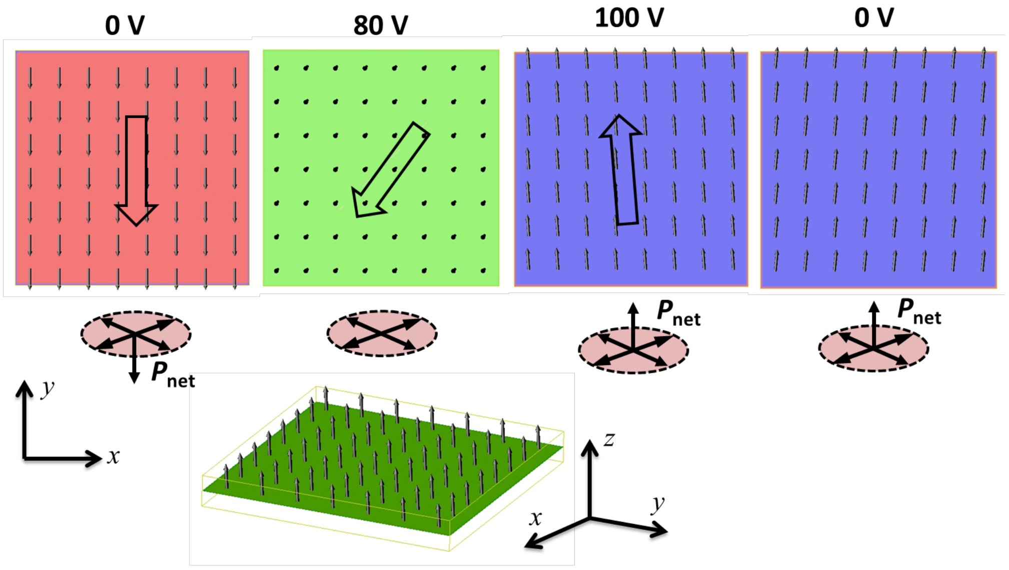

Figure S8 | Phase-field simulation of the magnetization distributions of NiFe with the PMN-PT polarization switching path from P_1^- to P_4, then to P_1^+.

S9. Mapping the planar magnetization distribution via magneto-optical Kerr microscope

The magnetization vector mapping images were obtained with data processing as follows: first, we measured both the longitudinal and transvers SKM images with each in-situ applied voltage during one scan. The images for the as grown state are shown in Fig. S9a, the “center value” of the Kerr signals are around 2007 mV and 1666 mV. Then we used an applied magnetic field to magnetize the sample and the fully saturated sample had a range of the SKM signals of 250 mV and 1120 mV for L and T signals, respectively. All signals subtract the center value, and then divided by the range value so that the whole image is normalized into a -1 to 1 range. As shown in Fig. S9, the images are the same in (a) and (b), only the color bar range is changed. Finally, the L and T values are combined in each pixel as x and y components of the vector to make the vector mapping image, as shown in Fig. S9c.

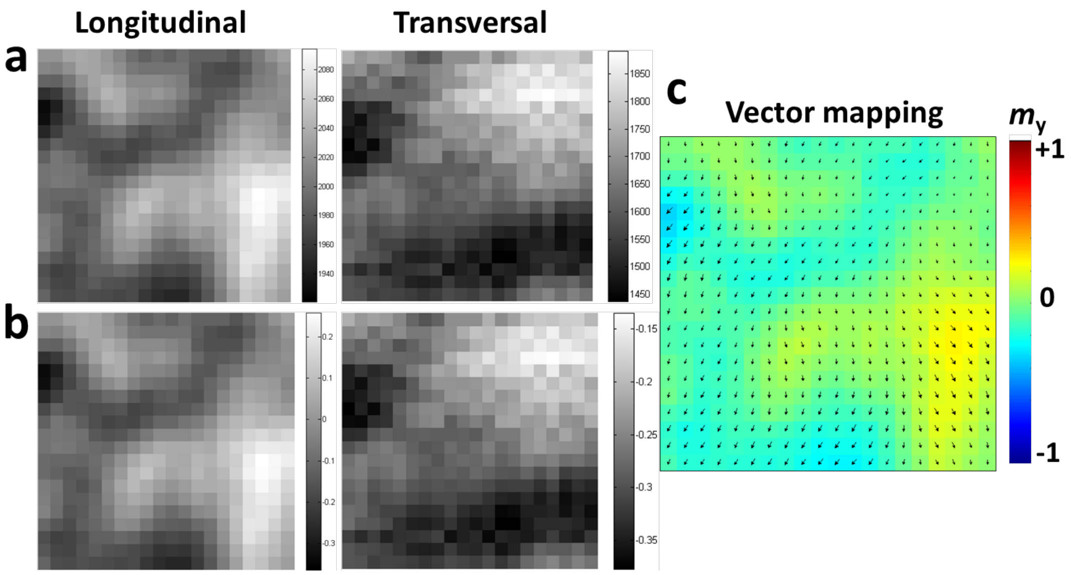

Figure S9 | SKM images: (**a**) original as-grown state SKM L and T images obtained in MOKE system. (**b**) Normalized L and T images for as-grown state. (**c**) mapping of the magnetization vectors for the as-grown state.

[1] Dawber, M., Rabe, K. M. & Scott, J. F. Physics of thin-film ferroelectric oxides. *Rev. Mod. Phys.* **77**, 1083 (2005).

[2] Johnson, M. T., Bloemen, P. J. H., Broeder, F. J. A. d. & Vries, J. J. d. Magnetic anisotropy in metallic multilayers. *Rep. Prog. Phys.* **59**, 1409 (1996).

[3] Saito, N., Fujiwara, H., Sugita, Y. A new type of magnetic domain structure in negative magnetostriction Ni-Fe films. *J. Phys. Soc. Jpn.* **19**, 1116-1125 (1964).

[4] Wang, J. J., Hu, J. M., Chen, L. Q. & Nan C. W., *Appl. Phys. Lett.*, **103**, 142413, (2013).
